# Compost, plants and endophytes versus metal contamination: choice of a restoration strategy steers the microbiome in polymetallic mine waste

**DOI:** 10.1186/s40793-023-00528-3

**Published:** 2023-10-07

**Authors:** Martina Kracmarova-Farren, Jakub Papik, Ondrej Uhlik, John Freeman, Andrea Foster, Mary-Cathrine Leewis, Courtney Creamer

**Affiliations:** 1 Department of Biochemistry and Microbiology, Faculty of Food and Biochemical Technology, University of Chemistry and Technology, Prague, Technicka 3, 166 28 Prague 6, Czech Republic; 2 Intrinsyx Environmental, Sunnyvale, CA 94085 USA; 3 U.S. Geological Survey, Menlo Park, CA USA; 4 Agriculture and Agri-Food Canada, Quebec Research and Development Centre, Quebec, QC Canada

**Keywords:** Mine tailings, Trace elements, Restoration, Endophyte, Compost, Microbial communities

## Abstract

Finding solutions for the remediation and restoration of abandoned mining areas is of great environmental importance as they pose a risk to ecosystem health. In this study, our aim was to determine how remediation strategies with (i) compost amendment, (ii) planting a metal-tolerant grass *Bouteloua curtipendula*, and (iii) its inoculation with beneficial endophytes influenced the microbiome of metal-contaminated tailings originating from the abandoned Blue Nose Mine, SE Arizona, near Patagonia (USA). We conducted an indoor microcosm experiment followed by a metataxonomic analysis of the mine tailings, compost, and root samples. Our results showed that each remediation strategy promoted a distinct pattern of microbial community structure in the mine tailings, which correlated with changes in their chemical properties. The combination of compost amendment and endophyte inoculation led to the highest prokaryotic diversity and total nitrogen and organic carbon, but also induced shifts in microbial community structure that significantly correlated with an enhanced potential for mobilization of Cu and Sb. Our findings show that soil health metrics (total nitrogen, organic carbon and pH) improved, and microbial community changed, due to organic matter input and endophyte inoculation, which enhanced metal leaching from the mine waste and potentially increased environmental risks posed by Cu and Sb. We further emphasize that because the initial choice of remediation strategy can significantly impact trace element mobility via modulation of both soil chemistry and microbial communities, site specific, bench-scale preliminary tests, as reported here, can help determine the potential risk of a chosen strategy.

**Supplementary Information:**

The online version contains supplementary material available at 10.1186/s40793-023-00528-3.

## Introduction

Sulfidic polymetallic mine tailings, or waste materials that accumulate after mineral extraction during mining activities, are residual solids from which resource elements (e.g., Ag, Mn, Pb, Zn) have been extracted by physical and/or chemical methods [1, 2]. Regardless of their environmental toxicity, they can host diverse microbial communities [3–6]. These communities participate in geochemical processes, such as the oxidative dissolution of sulfide minerals, which can lead to acid mine drainage (AMD) [7, 8]. The microbially catalyzed oxidation of metallic compounds can accelerate the release of trace elements into groundwater [9], and, thus, can pose a serious risk to people and all living parts of the ecosystem. Consequently, understanding the microbiome of these environments, and ways to manipulate it to prevent environmental harm, is of great importance.

Phytoremediation, or the use of plants and their associated microorganisms to remediate contaminated soils, is a common strategy for restoration of disturbed sites; for a review see Wang et al. [2], Sun et al. [10]. It is cost-effective, eco-friendly and suitable for difficult-to-access sites, such as remote historic mines. Different plant species have been shown to positively alter the physical and chemical attributes of mine tailings by increasing the amount of organic carbon and nitrogen [11, 12], while decreasing the mobility of trace elements by accumulation and adsorption to minerals and/or biological materials such as cells, biofilms, or exopolysaccharides [13–17]. In addition, it has been reported that the revegetation of different mine tailings affected the structure and diversity of their microbial communities [9, 18–21].

As mine tailings are some of the most hostile substrates for plant growth [22, 23], choosing plant species that can succeed in these unfavorable environments remains a challenge. Even the use of metal-tolerant species [24–27], which have the advantage of being adapted to high concentrations of trace elements, or hyperaccumulators [28, 29] can be limiting in the terms of biomass yield. For that reason, the use of organic amendments for improved phytoremediation offers the promise of a more effective restoration of metal-contaminated sites [30]. In addition to promoting plant growth, amendments such as compost can reduce the phytoavailability of toxic trace elements in soil [13, 31–33]. Moreover, in contrast to chemical fertilizers, compost amendment is eco-friendly and cost-effective and can improve soil health metrics by increasing water-holding capacity, pH, and microbial activity [13, 14, 34–36] and thus, can accelerate the restoration of abandoned mining areas.

Another way of enhancing plant biomass production is by inoculating plants with beneficial endophytes. Endophytes are microorganisms that live inside plants for at least part of their life cycle [37]. The endophytes can produce phytohormones and siderophores, fix atmospheric nitrogen, solubilize inorganic phosphorus, or protect the host plant against biotic and abiotic stresses; for a review, see Papik et al. [38]. In addition, some endophytic bacteria have been shown to transform toxic trace elements into non-toxic and/or bioavailable forms, thereby resulting in increased tolerance to and accumulation of trace elements in different plant species [29, 39–41]. While some studies have shown that endophyte inoculation or compost amendment enhanced the phytoremediation of metal-contaminated soils, including mine tailings [13, 42–44]), the monitoring of how these approaches influence the interactions between microbial communities, trace element mobility, and soil health metrics during compost- or endophyte-assisted mine waste phytoremediation has not been extensively studied.

In this study, we analyzed the microbial communities in soil, compost, and plant roots from a microcosm experiment with a fully factorial design aiming to evaluate how different restoration approaches: (i) compost amendment, (ii) planting a metal-tolerant grass *Bouteloua curtipendula*, and (iii) its inoculation with beneficial endophytes, influenced the microbiota and chemical properties of mine tailings. We hypothesized that (i) our approaches would act in synergy and increase microbial diversity in the mine tailings, (ii) the fingerprint of each strategy would be detectable in microbial community structure, and (iii) the resulting changes in tailings microbiota would be associated with changes in soil chemistry. This study specifically describes the interactive effects between tailings microbiota, trace element mobility, and organic matter content which provides a novel link between biotic and abiotic mine waste responses to different restoration approaches and combinations.

## Materials and methods

### Experimental design

**Fig. 1:**
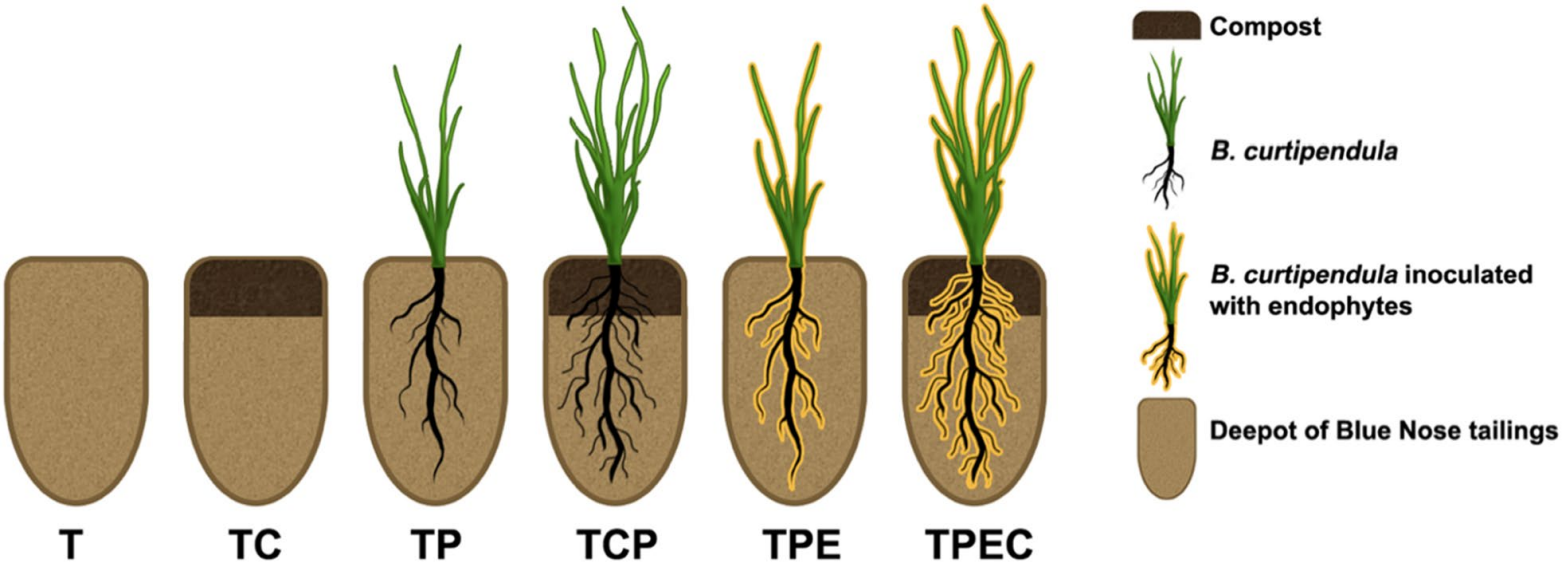
Schematic representation of the pot experiment. The treatments included: tailings (T), tailings with added compost (TC), tailings with a plant (TP), tailings with a plant and added compost (TPC), tailings with a plant inoculated with endophytes (TPE) and tailings with a plant inoculated with endophytes and compost (TPEC)

For this pot experiment, we selected sulfidic polymetallic mine tailings originating from the abandoned Blue Nose mine near Tucson, Arizona (31.44782, − 110.73293, 1600 m elevation) with pH of 3.5 and high content of potentially toxic trace elements (As, Cd, Cu, Mn, Pb, Sb, Tl, Zn). Specifically, concentrations of As and Pb were 10–20 times soil remediation levels [45], and Cd, Cu, Mn, Pb, Sb, and Zn exceeded water quality standards for people and wildlife [46], as we previously reported [47]. General characterization and total trace element content of pre-treatment materials are summarized in Additional file 1: Table S1. *Bouteloua curtipendula* was selected because it is metal-tolerant, native to the US, and was found at the sampling site [48]. In total, there were six treatments: tailings only (T, control, n = 8), tailings with added compost (TC, n = 8), tailings with a plant (TP, n = 16), tailings with a plant and added compost (TPC, n = 9), tailings with a plant inoculated with endophytes (TPE, n = 16), and tailings with a plant inoculated with endophytes and added compost (TPEC, n = 9) (Fig. 1). Due to previously observed low germination (c.a. 10%) and high mortality rates (c.a. 50%) of *B. curtipendula* in tailings without amendments [47], twice as many pots were planted in treatments without compost (n = 16) and one extra pot was planted in TPC treatment (n = 9) compared to the controls (n = 8) to ensure a sufficient number of biological replicates.

### Plant cultivation

Pots (Deepots™; 5 × 18 cm, Stuewe & Sons, Tangent, OR USA) were filled with 240 g of mine tailings mixed with dolomite (35.34 mg/g, corresponding to a rate of roughly 40 tonnes/hectare) to raise their pH to 5.1 (further referred to as the “initial mine tailings”), which was necessary to support plant growth, and wetted with 15 ml of deionized water. *B. curtipendula* seeds were either coated with microbial coculture of ten beneficial endophytes (TPE and TPEC) (Additional file 1: Table S2) or sterile medium (TP and TPC). The coating procedure was performed as follows: the endophyte coculture was obtained via cultivation in a liquid N-limited Rennie medium [49] under aerobic conditions to an optical density of 0.5 at 600 nm. The coculture was subsequently sprayed onto *B. curtipendula* seeds (50 mL/30.5 g of seed for 1 min) and coated seeds were dried for 3 days at 25 °C. Control seeds were processed analogously but coated with sterile medium. Fifteen seeds were directly sown into the mine tailings in each pot the day after the pots were filled with tailings. In case of compost treatments (TC, TPC, TPEC), wet municipal compost was added (15.3 mg/g, corresponding to a rate of roughly 60 tonnes/hectare) to sowed pots, forming an additional layer above the tailings. Seeds had higher germination rate when planted with compost: about 50% of the seeds germinated when planted with compost, but only 30% of the seeds germinated without compost. In the absence of a compost layer, the seeds had similar germination regardless of whether they were coated with endophytes or not [50]). The pots were thinned upon seedling germination to obtain 1–2 seedlings per pot. In total, the pots were cultivated for two months in Deepots™ with nylon mesh lining in indoor growth chambers (one chamber per treatment), each of which was equipped with a fan enabling continuous air circulation and a LED grow light (HIGROW™ 600W) placed approximately 40 cm above the Deepot racks. The lighting in chambers followed a 12 h diurnal cycle (12 h on; 12 h off). Pots were regularly watered using the automatic gravity-fed drip irrigation system to maintain a target of 55% water holding capacity. For treatments without compost (TP and TPE), 3 out of 16 pots did not germinate or all its seedlings died.

### Sample collection, processing, and chemical analyses

After 2 months of plant growth, the pots were destructively harvested over the course of three days to process and subsample the mine tailings, compost, and plants for further analyses. Due to a large number of samples that required immediate processing upon harvest, the pots corresponding to TP and TPC treatments were harvested after 56 days of plant growth, TPE and TPEC treatments on day 57, and T and TC treatments on day 58. To harvest the pots, first, if the compost layer was present, it was separated from the mine tailings. Subsequently, the *B. curtipendula* roots were gently removed from the mine tailings and washed with deionized water. Roots were stored for two days at 4 °C and then, together with seeds, surface-sterilized by vigorous agitation in 70% ethanol for 3 min, followed by agitation in 2.5% (v/v) sodium hypochlorite (NaOCl) for 5 min as described by Barra et al. [51]. Immediately after the surface sterilization, the roots were washed with sterile deionized water three times for 5 min per wash cycle. One hundred µL of the final wash solutions were spread on Luria Bertrani (LB) agar plates and incubated at 28 °C for 5 days to check for sterility. Surface-sterilized roots and seeds were then stored at − 80 °C until grinding, which was performed aseptically in liquid nitrogen in a ceramic mortar using a pestle. Due to low biomass quantities, only the roots from TPC and TPEC were used for the analysis of endophytic microbial communities, and the roots from pots within a treatment were combined to enable at least three replicates to provide the minimum of 20 mg of root biomass for the DNA extraction.

Harvested mine tailings and compost layers were homogenized using a sterile plastic spoon and approximately 2 g were subsampled and stored at − 20 °C for DNA extraction. The remaining homogenized compost was air-dried for 14 days, and homogenized tailings were oven-dried at 40 °C for 7–8 days. The initial mine tailings, compost, and seeds inoculated with either the endophytic coculture or sterile medium were processed analogously.

Mine tailings subsamples were subjected to the analysis of basic soil properties, including pH, carbon and nitrogen content, and elemental composition [details were presented in Creamer et al. [52]. The pH was measured in deionized water [1:5 solid: solution ratio (w/v)] after agitation for 15 min at 110 rev/min and settling for 10 min. Fifteen grams of mine tailings were ground to a fine powder (< 105 µm) in a sintered corundum (99.7% Al_2_O_3_) planetary ball mill (25 min at speed 7; Fritsch pulverisette™ 5, CA, USA) and analyzed for total nitrogen and carbon in a CN analyzer (Carlo-Erba™, CE Elantech, Lakewood NJ). Organic carbon was analyzed using the CN analyzer after overnight fumigation with 12 M HCl [53]. To quantify 51 water-soluble elements, the ground mine tailings were agitated with deionized water in a 1:30 (m/v) ratio for 2 h and analyzed by using inductively coupled plasma optical emission spectroscopy (ICP-OES) or mass spectrometry (ICP-MS). Analyses were conducted by AGAT Labs (Canada) under contract to the U.S. Geological Survey Mineral Resources Program (Analytical Chemistry Division, Denver, CO) with QA/QC as described in Creamer et al. [52].

### DNA extraction and quantification

Total DNA was extracted from 700 mg of mine tailings, 500 mg of compost, and 20 mg of surface-sterilized roots using the FastDNA SPIN kit for soil (MP Biomedicals, Ohio, USA) following the manufacturer’s protocol with a few modifications to enhance DNA yield: increased time of homogenization (15 min) and air-drying the samples in a laminar flow hood (10 min) prior to DNA elution [20]. DNA samples were purified and concentrated using the DNA Clean and Concentrator kit (Zymo Research, Irving, CA, USA). DNA concentration was determined using a PicoGreen dsDNA Assay Kit (Thermo Fisher Scientific Technologies, Wilmington, DE) and normalized to 15 ng/µL per sample prior to the library preparation for amplicon sequencing.

The abundance of bacterial and fungal marker genes in compost and tailings was quantified with qPCR on purified DNA (diluted to 2 ng/µL). The bacterial community abundance (16S rRNA gene) was assessed using primers and conditions from Fierer et al. [54]: forward Eub338 (5′-GCTGCCTCCCGTAGGAGT-3′) and reverse Eub518 (5′-ATTACCGCGGCTGCTGG-3). Fungal community abundance (28S rRNA gene) was assessed with primers and conditions detailed in White et al. [55]: cTW13 (5′-CGTCTTGAAACACGGACC-3′) and TW14 (5′-GCTATCCTGAGGGAAACTTC-3′). Each 10 µL PCR reaction for 16S and 28S rRNA contained: 5 µL of KAPA SYBR FAST qPCR Master Mix (2X) Universal (0.02 U/μL, KAPA Biosystems, Boston, USA), 0.2 µL of Low Rox, 0.2 µL of each forward and reverse primers (10 µM), 2 µL of template DNA (2 ng/µL) and PCR-grade water.

### 16S rRNA gene and ITS2 region amplicon sequencing

The V4-V5 hypervariable region of the 16S rRNA gene was amplified using the 515 forward (5′-GTGYCAGCMGCNGCGG-3′) and 926 reverse primers (5′-CCGYCAATTYMTTTRAGTTT-3′) [56]. ITS2 region was amplified using 5.8S_Fun forward primer (5′-AACTTTYRRCAAYGGATCWCT-3′) and ITS4_Fun reverse primer (5′-AGCCTCCGCTTATTGATATGCTTAART-3′) [57].

Mine tailings and compost DNA samples were amplified using a two-step PCR process. The first 15 µL reaction contained: 0.02 U/µl KAPA HiFi HotStart ReadyMix (Kapa Biosystems, USA), 0.3 µM of each primer (Sigma-Aldrich, USA), ~ 10 ng of template DNA and PCR grade water (Sigma-Aldrich, USA). The cycling conditions were as follows: an initial DNA denaturation for 5 min at 95 °C, 25–28 cycles of 20 s at 98 °C, 15 s at 56 °C (16S rRNA) or 50 °C (ITS), 15 s at 72 °C, and final extension for 5 min at 72 °C [58]. A volume of 0.5 µL of the PCR product was used as a template in a second PCR with the same primers containing internal barcodes and sequencing adapters [59]. This round of PCR was performed analogously as before, except that the final reaction volume was 25 µL, the concentration of each primer was 1 mM, and the number of cycles and annealing temperature were decreased to 8–10 and 50 °C, respectively.

For root DNA samples, ITS2 region amplicons were prepared using the same 2-step PCR procedure as described above for the mine tailings and compost samples. For 16S rRNA gene amplicon preparation, each sample was amplified using three rounds of PCR. First, peptide nucleic acids were used to prevent the amplification of mitochondrial (mPNA) and plastid (pPNA) DNA [60]. This 15 µL reaction contained: 0.3 µM of each PNA probe: mPNAs (5′-GGCAAGTGTTCTTCGGA-3′) and pPNAs (5′-GGCTCAACCCTGGACAG-3′) (PNA Bio, Thousand Oaks, CA), 0.02 U/µl of KAPA HiFi HotStart ReadyMix (Kapa Biosystems, USA), 0.3 µM of the 515 forward primer, 0.3 µM of 1068 reverse primer (5′-CTGRCGRCRRCCATGCA-3′, Sigma-Aldrich, USA), 10 ng of template DNA and PCR grade water (Sigma-Aldrich, USA) [61]. The temperature cycling conditions were as follows: initial DNA denaturation at 95 °C for 5 min, 25–30 cycles of 20 s at 98 °C, 15 s at 75 °C (annealing of the PNAs), 15 s at 50 °C, 15 s at 72 °C, and final extension at 72 °C for 5 min. Each sample was prepared in 9 copies that were pooled together and separated by electrophoresis on 1.5% agarose gel. The fragments corresponding to the size of 550 bp were excised from the gel and purified using a Zymoclean Gel DNA Recovery Kit (ZYMORESEARCH, USA). 0.5 µL of the purified PCR product was used as a template in the same 2-step PCR process as described above for the mine tailings and compost.

Amplicons were then purified with SPRI magnetic beads (Beckman Coulter, USA) according to the manufacturer’s instructions. The concentration of each purified sample was measured using a Picogreen assay for dsDNA (Thermo Fisher Scientific Technologies, Wilmington, DE) following the manufacturer’s protocol. To identify potential sequencing errors during data processing, mock community DNA standards (ZymoBIOMICS Microbial Community DNA Standard, Zymo Research, Irvine, CA) were used and subjected to the same procedures as the mine tailings, compost, and root DNA samples. All further downstream analyses, including the finalization of library preparation and sequencing, were performed at the Core Facility for Nucleic Acid at the University of Alaska, Fairbanks as follows: purified amplicons were pooled in equimolar concentrations using Sequal Prep Kit (Thermo Fisher Scientific Technologies, Wilmington, DE), and the final quality and concentration of the library were determined via NEBNext Library (New England BioLabs, Ipswich, MA). Libraries were spiked with PhiX (15%) and followed standard Illumina denature and dilute protocols. 10 pmol of amplicon libraries were then loaded and sequenced using the Illumina MiSeq V3 reagent kit.

### Data processing and statistical analyses

Sequence data were processed using the DADA2 pipeline [62] in R (v.4.1.0) [63] with a few modifications. The primer sequences were removed if found present, otherwise the whole read was discarded. 16S rRNA gene sequences were filtered and trimmed using the following parameters: trimLeft = c(0, 0), maxN = 0, maxEE = 2, truncQ = 2. The reads were truncated to 247 and 189 bases for forward and reverse reads, respectively. ITS region sequences were filtered using the same parameters, but no truncation was performed. 16S rRNA sequences were merged if they differed by only a single base and ITS2 region sequences were merged if they differed by up to two bases, bioinformatic steps were chosen based on analysis of the mock community. To create the database of amplicon sequence variants (ASVs), taxonomy was assigned using the silva_nr_v132_train_set.fa.gz database [64] and the UNITE database [65] for 16S rRNA gene and ITS region, respectively. All further statistical analyses were performed in R within the *phyloseq* [66], *vegan* [67] and *DESeq2* [68] packages. Differences at *p* ≤ 0.05 were considered statistically significant. All graphical outputs were created using *ggplot2* [69] package.

To describe alpha diversity of the microbial communities, we calculated the Shannon diversity index of microbial communities [70] and tested how different treatments (T, TC, TP, TPC, TPE, TPEC) influenced microbial diversity using a Pairwise Wilcoxon rank sum test. The resulting *p* values were adjusted using a false discovery rate (FDR) method. The sequence datasets were rarefied to the smallest sample size and the relative abundance of 16S rRNA and ITS ASVs at the phylum level were displayed in bar plots to compare the phyla representation across treatments. To test the influence of (i) compost amendment, (ii) endophyte inoculation, (iii) presence of *B. curtipendula*, as well as the interaction of these variables on microbial community structure at an ASV level, the data were Hellinger-transformed [71], and permutational multivariate analysis of variance (PERMANOVA, 999 permutations) based on Bray–Curtis dissimilarity was used [72]. In addition, a Pairwise PERMANOVA was conducted to compare microbial community structure between individual treatments with the resulting *p* values being adjusted using a FDR method. To investigate which microbial genera had significantly different abundance between T and either TC, TP, TPC, TPE, or TPEC, we used differential expression analysis (*DESeq2* package) on non-transformed and non-rarefied datasets merged at the genus level [68]. In total, five pairwise comparisons were performed: (i) T vs TP, (ii) T vs TPE, (iii) T vs TC, (iv) T vs TPC, and (v) T vs TPEC. The *lfcShrink* function was applied to shrink logarithmic fold change values. We established a logarithmic fold (Log twofold) change threshold of 1.2 and a FDR of 1% as criteria for determining statistical significance. The results of differential abundance analyses were presented using heatmaps with a dendrogram constructed using Ward’s hierarchical clustering (Euclidean distance, genus level), with missing values being replaced with zeroes.

In order to compare the values of water-extractable trace elements in treated tailings against control, a Pairwise Wilcoxon rank sum test was performed with *p* values being adjusted using a FDR method. To further explore how measured chemical properties correlated with microbial community structure, the chemical parameters were fitted onto a Bray–Curtis-based NMDS ordination using the envfit function from the *vegan* package [67].

Differences in 16S rRNA or 28S rRNA gene copy number, and the ratio of bacterial to fungal gene copy number (F:B ratio) in compost and tailings samples collected after 2 months of plant growth were analyzed with a Kruskal–Wallis test using treatment (no treatment, compost, plant, or plant + endophyte addition) as the factors. Differences between treatments were further tested using a Pairwise Wilcoxon rank sum test, and corrected for multiple comparisons using a FDR correction.

## Results

### Chemical properties of the mine tailings

The detailed results of the mine tailings analyses are described in Creamer et al. [52], however we briefly summarize those finding here (Table 1). Blue Nose mine tailings contained several potentially toxic water-extractable trace elements at high concentrations: As, Cd, Cu, Mn, Pb, Sb, and Zn, which exceeded water quality criteria for people and wildlife [46], as well as low concentrations of organic carbon and nitrogen (Table 1). As there were significant differences between control (T) and all treatments for several measured chemical parameters (*p*_adj_ ≤ 0.05, Pairwise Wilcoxon rank sum test; Table 1), the associations between microbial community structure and tailings chemistry under different treatments were subsequently investigated.

**Table 1:** Water-extractable trace elements and soil health metrics of the mine tailings (mean ± standard deviation)

	T_in_	T	TC	TP	TPC	TPE	TPEC
As (ng/g)	11	75 ± 19	56 ± 21	86 ± 20	76 ± 27	74 ± 17	66 ± 19
Cd (ng/g)	2160	706 ± 46	669 ± 17	701 ± 34	727 ± 29	696 ± 29	672 ± 24
Co (ng/g)	798	147 ± 9	145 ± 5	137 ± 12	155 ± 8	141 ± 7	145 ± 5
Cu (ng/g)	1210	557 ± 59	639 ± 109	**296 ± 121**	**754 ± 50**	**657 ± 106**	**830 ± 67**
Mn (μg/g)	455	68 ± 8	61 ± 9	71 ± 6	**81 ± 10**	68 ± 5	62 ± 4
Pb (μg/g)	104	73 ± 4	69 ± 4	72 ± 4	69 ± 4	74 ± 5	68 ± 2
Sb (ng/g)	137	600 ± 47	**690 ± 29**	**548 ± 34**	**693 ± 43**	608 ± 34	**703 ± 44**
Zn (μg/g)	215	21 ± 2	21 ± 1	21 ± 2	22 ± 1	20 ± 1	21 ± 1
pH	5.11	6.63 ± 0.03	6.7 ± 0.06	6.68 ± 0.06	6.69 ± 0.04	6.65 ± 0.06	**6.75 ± 0.05**
Total nitrogen (mg/g)	0.08	0.14 ± 0.04	**0.16 ± 0.01**	**0.12 ± 0.01**	**0.17 ± 0.01**	0.12 ± 0.02	**0.16 ± 0.01**
Inorganic carbon (mg/g)	4.70	3.99 ± 0.31	3.79 ± 0.22	3.66 ± 0.29	3.73 ± 0.28	3.90 ± 0.34	3.67 ± 0.18
Organic carbon (mg/g)	2.00	2.00 ± 0.07	**2.28 ± 0.11**	**2.09 ± 0.07**	**2.44 ± 0.20**	**2.10 ± 0.08**	**2.48 ± 0.06**

Pre-treatment materials: initial tailings (T_in_). Treatments: tailings (T, n = 8), tailings with added compost (TC, n = 8), tailings with a plant (TP, n = 13), tailings with a plant and added compost (TPC, n = 8), tailings with a plant inoculated with endophytes (TPE, n = 11) and tailings with a plant inoculated with endophytes and added compost (TPEC, n = 7). Values corresponding to treatments that significantly differed from control, i.e., T, (*p*_*adj*_ ≤ 0.05, Pairwise Wilcoxon rank sum test) are underlined and shown in bold. Full data sets available for download from Creamer et al. [52] and Creamer et al. [73] (10.5066/P9M2JW70 and 10.5066/P99OYEXQ)

### Microbial abundance

We quantified bacterial and fungal populations (i.e., copy numbers of 16S rRNA and 28S rRNA genes, respectively) separately in compost and tailings layers (Additional file 1: Fig. S1). In compost, fungal gene copy number was significantly different (*p* = 0.02, Kruskal–Wallis) across treatments after 2 months of plant growth and further testing revealed that fungal gene copy number was significantly higher in the TPC treatment compared to TPEC treatment (*p*_adj_ = 0.046, Pairwise Wilcoxon rank sum test). In tailings, the ratio of fungal:bacterial abundance was significantly increased in treatments with added compost after 2 months of plant growth (*p*_adj_ < 0.05, Pairwise Wilcoxon rank sum test). There were no significant differences in bacterial and fungal gene copy numbers in tailings among treatments (*p*_adj_ > 0.05, Pairwise Wilcoxon rank sum test).

### Microbial diversity in compost, *B. curtipendula* roots, and tailings

**Fig. 2:**
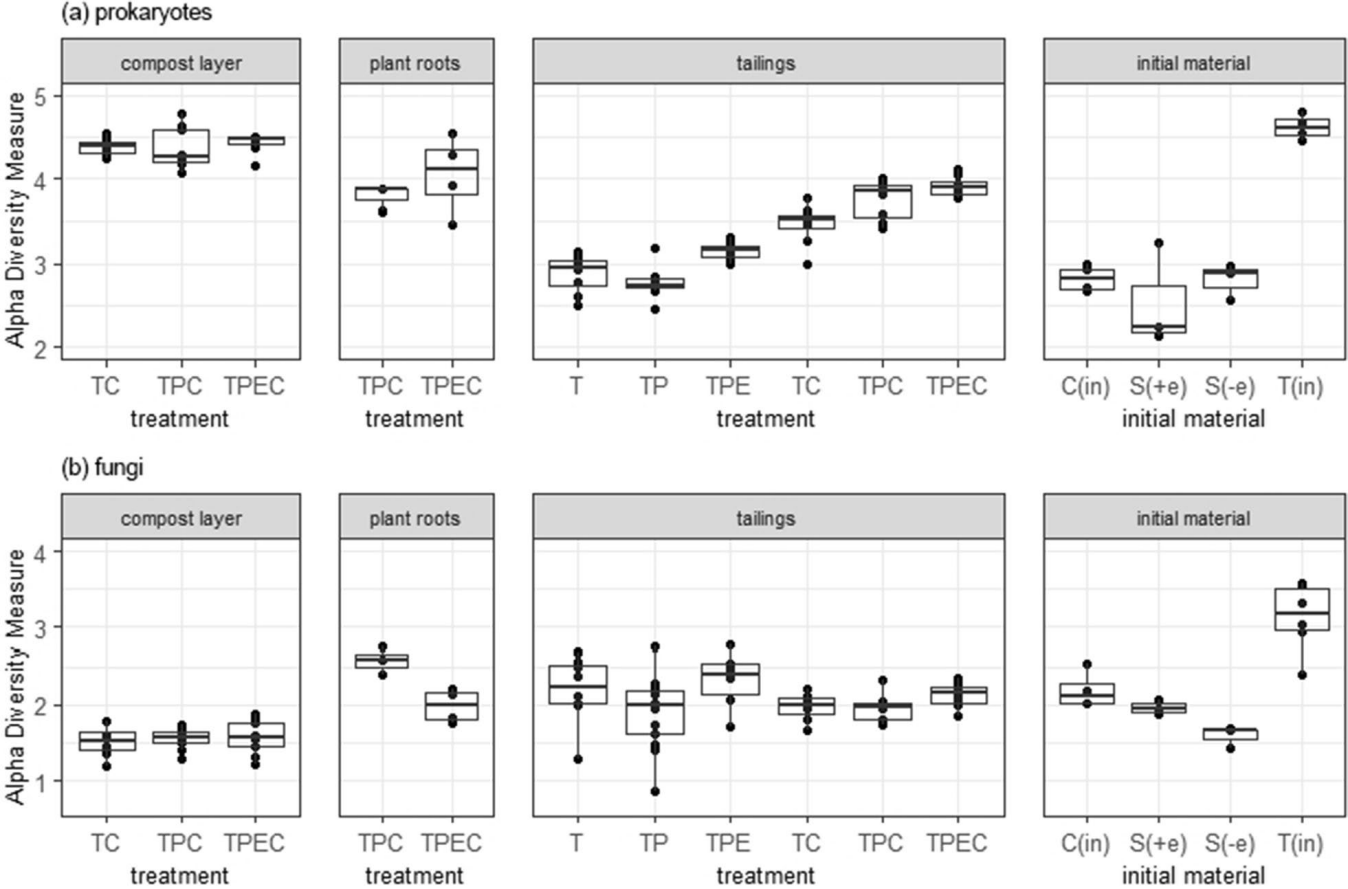
Prokaryotic (**a**) and fungal (**b**) diversity in compost, roots, and tailings incubated under different treatments: tailings (T), tailings with added compost (TC), tailings with a plant (TP), tailings with a plant and added compost (TPC), tailings with a plant inoculated with endophytes (TPE) and tailings with a plant inoculated with endophytes and added compost (TPEC). Initial materials included initial tailings (T_in_), initial compost (C_in_), seeds inoculated with beneficial endophytes (S_+e_), and seeds treated with sterile medium (S_-e_)

In total, 3,532,593 16S rRNA gene sequences and 3,017,378 ITS2 region sequences were obtained. ASVs assigned to mitochondria at the family level or to chloroplast at the order level were discarded from the 16S rRNA ASV dataset, accounting for 1.8% sequences in total. The remaining datasets were rarefied to the smallest sample size: 4,400 and 3,200 reads for 16S rRNA and ITS, respectively, which resulted in 2,396 unique prokaryotic taxa and 615 unique fungal taxa. Shannon diversity index (Fig. 2) showed that alpha diversity of both prokaryotic and fungal communities was significantly higher in the initial mine tailings (T_in_) when compared to the tailings (T, TP, TPE, TC, TPC, TPEC) collected after two months of plant growth (*p*_*adj*_ < 0.01 for prokaryotes and *p*_*adj*_ < 0.05 for fungi, Pairwise Wilcoxon rank sum test, Additional file 1: Table S3). Prokaryotic diversity was significantly higher in the tailings of all treatments except for TP when compared to the control T (*p*_*adj*_ < 0.01, Pairwise Wilcoxon rank sum test), and the highest diversity increase in tailings was observed when compost amendment, planting and endophyte inoculation were all combined (TPEC). Fungal diversity in the tailings of all treatments did not significantly differ from the control T (*p*_*adj*_ > 0.05, Pairwise Wilcoxon rank sum test), but TPE treatment resulted in significantly higher diversity compared to TP, TC, and TPC treatments (*p*_*adj*_ < 0.05, Pairwise Wilcoxon rank sum test, Additional file 1: Table S3). Microbial diversity in the initial compost (C_in_) also significantly differed from the compost samples collected after two months of plant growth; prokaryotic diversity was significantly lower while fungal diversity was significantly higher in C_in_ vs TC, TCP, and TPEC (*p*_*adj*_ < 0.05, Pairwise Wilcoxon rank sum test, Additional file 1: Table S3). In contrast to tailings, microbial diversity in compost did not significantly differ across the treatments, and endophytic diversity in roots of *B. curtipendula* was not significantly associated with endophyte inoculation (*p*_*adj*_ > 0.05, Pairwise Wilcoxon rank sum test).

### Microbial community structure in compost, roots, and tailings under different treatments

**Fig. 3:**
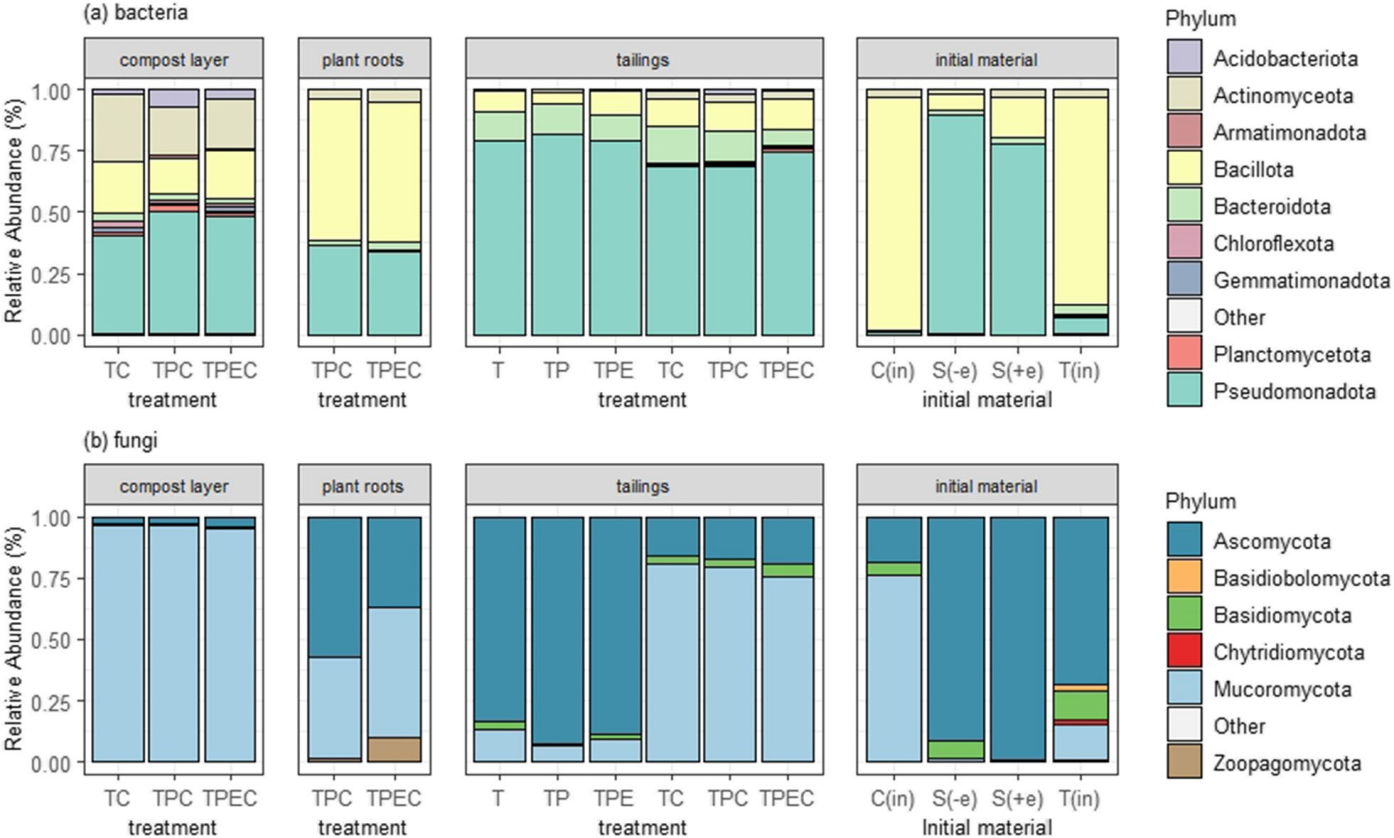
Relative abundance of bacterial (**a**) and fungal (**b**) phyla in compost, roots, and tailings across the treatments: tailings (T), tailings with added compost (TC), tailings with a plant (TP), tailings with a plant and added compost (TPC), tailings with a plant inoculated with endophytes (TPE) and tailings with a plant inoculated with endophytes and added compost (TPEC). Initial materials include initial compost (C_in_), seeds without and with endophytes coating (S_-e_, and S_+e_, respectively), and initial tailings (T_in_). Phyla with relative abundance lower than 0.1% are grouped and labeled as “Other”

In total, 24 prokaryotic and ten fungal phyla were detected in the samples of compost, roots, tailings, and initial materials. Nine bacterial and six fungal phyla had a higher relative abundance than 0.1% (Fig. 3). The largest differences in the distribution of bacterial phyla were observed between the initial tailings and compost materials (T_in_, C_in_) and those collected at the end of the pot experiment. At project initiation, T_in_ and C_in_ were dominated by Bacillota, and at project harvest (after 56 days; 57 days; and 58 days in the case of TP and TPC; TPE and TPEC; and T and TC treatments, respectively) all tailings were dominated by Pseudomonadota. The distribution of dominant fungal phyla was notably different among treatments without compost (T, TP, and TPE), which were largely dominated by the reads of Ascomycota, and with compost (TC, TPC, and TPEC), which were largely dominated by Mucoromycota (Fig. 3).

Both prokaryotic and fungal community structure in the mine tailings were significantly associated with compost amendment, the presence of *B. curtipendula*, and the interaction of these two factors (PERMANOVA at ASV level, Table 2). The changes in prokaryotic community structure were also associated with endophyte inoculation and its interaction with compost amendment. These results are consistent with the pairwise comparison of individual treatments, which showed significant differences in prokaryotic community structure between all the treatments (*p*_*adj*_ < 0.001, Pairwise PERMANOVA, Additional file 1: Table S4). Fungal community structure also significantly differed between treatments except for TC vs TPC, and TPC vs TPEC. Of all the variables tested, compost amendment explained most of the variation in microbial community structure: 39% in prokaryotic and 47% in fungal communities (PERMANOVA, Table 2). In particular, the highest variability in prokaryotic community structure was observed between TC vs TPE, TP vs TPEC, and TPE vs TPEC (R^2^ = 0.60, R^2^ = 0.65, and R^2^ = 0.67, respectively, Pairwise PERMANOVA, Additional file 1: Table S4). The highest variation in fungal community structure was observed between T vs TC, TPC vs TPE, TPE vs TPEC, and TC vs TPE (R^2^ = 0.58, R^2^ = 0.58, R^2^ = 0.58, and R^2^ = 0.61, respectively, Pairwise PERMANOVA, Additional file 1: Table S4). Both endophyte inoculation and the presence of *B. curtipendula* were significantly associated with the prokaryotic community structure in the compost sampled from amended pots, while no significant associations were found in the structure of compost fungal communities (Table 2). Finally, the fungal and prokaryotic endophytic communities in the roots of *B. curtipendula* were not found to be significantly associated with any treatment (PERMANOVA, Table 2).

**Table 2:** The association of compost amendment, endophyte inoculation, and the presence of *B. curtipendula* with microbial community structure in tailings, compost, and roots of *B. curtipendula* (PERMANOVA)

	(a) prokaryotes			(b) fungi		
	Endophyte inoculation	Compost amendment	Presence of *B. curtipendula*	Endophyte inoculation	Compost amendment	Presence of *B. curtipendula*
TAILINGS*	***p* = 0.001**	***p* = 0.001**	***p* = 0.003**	*p* = 0.095	***p* = 0.001**	***p* = 0.001**
	R^2^ = 0.07410	R^2^ = 0.39263	R^2^ = 0.03569	R^2^ = 0.01501	R^2^ = 0.46620	R^2^ = 0.06405
COMPOST	***p* = 0.001**	–	***p* = 0.001**	*p* = 0.565	–	*p* = 0.621
	R^2^ = 0.11744	–	R^2^ = 0.09765	R^2^ = 0.03312	–	R^2^ = 0.02535
ROOTS	*p* = 0.21	–	–	*p* = 0.416	–	–
	R^2^ = 0.21945	–	–	R^2^ = 0.15093	–	–
*Significant interaction of:						
(a) prokaryotes						
Compost amendment x Endophyte inoculation (R^2^ = 0.07614; ***p* = 0.001**), and Compost amendment x Presence of *B. curtipendula* (R^2^ = 0. 07614; ***p* = 0.001**)						
(b) fungi						
Compost amendment x Presence of *B. curtipendula* (R^2^ = 0.04570; ***p* = 0.001**)						

Significant *p* values (p ≤ 0.05) are underlined and shown in bold

**Fig. 4:**
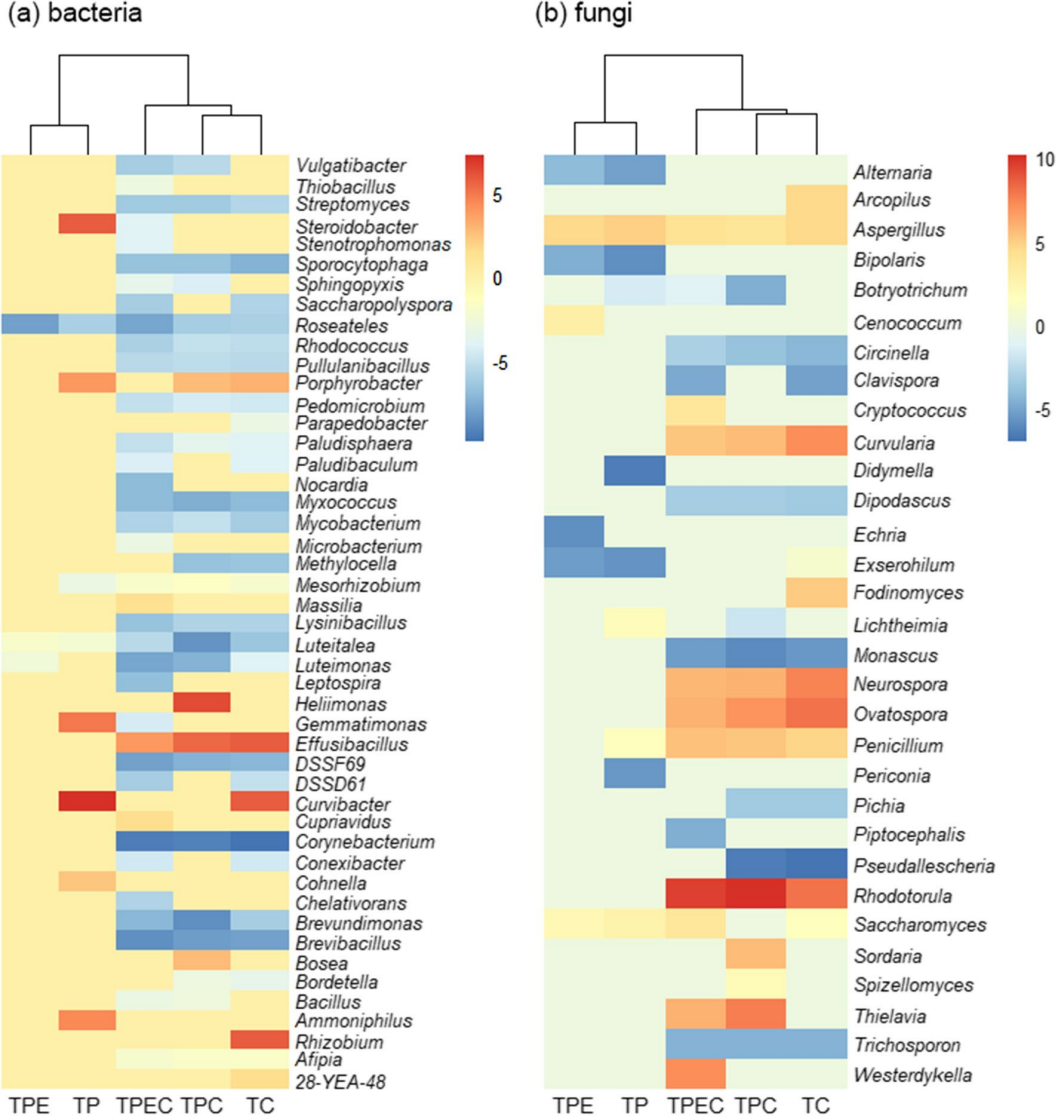
Heatmap of Log twofold change values representing significantly enriched bacterial (**a**) and fungal (**b**) genera across treatments: tailings with added compost (TC), tailings with a plant (TP), tailings with a plant and added compost (TPC), tailings with a plant inoculated with endophytes (TPE) and tailings with a plant inoculated with endophytes and added compost (TPEC) vs tailings only (T, control) as revealed by differential abundance analysis. Genera that were significantly more abundant in T are represented by a positive Log twofold change, while genera that were significantly more abundant in either TP, TC, TPE, TPC, or TPEC vs T are represented by a negative Log twofold change

Differential abundance analysis using DESeq2 revealed that 36 bacterial and 16 fungal genera were enriched in at least one of the treatments TC, TP, TPE, TPC, and TPEC when compared to T (*p*_*adj*_ ≤ 0.01, Fig. 4). The hierarchical clustering of obtained Log twofold change values, as shown in Fig. 4, revealed two main clusters: the grouping of treatments with compost (TPC, TC, TPEC) and treatments without compost (TP and TPE). When we compared the individual treatments vs control (T), we found that bacterial genera (Fig. 4a) were primarily enriched in treatments with compost, while the TPEC treatment resulted in the highest number of enriched genera (33) (Fig. 4a).

### Changes in tailings chemical properties related to microbial community structure

**Fig. 5:**
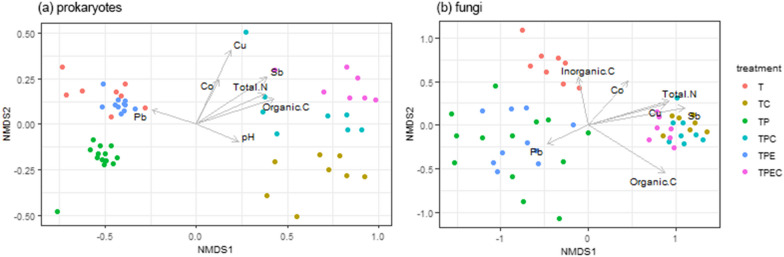
Non-metric multidimensional scaling (NMDS) of prokaryotic (**a**, stress = 0.077) and fungal (**b**, stress = 0.13) communities in tailings using Bray–Curtis distances and subsequent fitting of environmental variables (*p* ≤ 0.05) significantly associated with the distribution of samples in the ordination space. Treatments: tailings (T), tailings with added compost (TC), tailings with a plant (TP), tailings with a plant and added compost (TPC), tailings with a plant inoculated with endophytes (TPE) and tailings with a plant inoculated with endophytes and added compost (TPEC)

Statistically significant correlations (NMDS with subsequent fitting of environmental variables, *p* ≤ 0.05) between the chemical properties of the mine tailings samples and microbial community structure are displayed in Fig. 5. The ordination plots show a clear separation of both prokaryotic and fungal communities in the mine tailings along the first axis, forming two main clusters based on the presence (TC, TPC, TPEC) or absence of compost (T, TP, TPE). The content of organic carbon (R^2^ = 0.63 for prokaryotes, R^2^ = 0.53 for fungi, *p* ≤ 0.05), total nitrogen (R^2^ = 0.53 for prokaryotes, R^2^ = 0.47 for fungi, *p* ≤ 0.05), Sb (R^2^ = 0.68 for prokaryotes, R^2^ = 0.63 for fungi, *p* ≤ 0.05), and Cu (R^2^ = 0.64 for prokaryotes, R^2^ = 0.42 for fungi, *p* ≤ 0.05) were identified as the factors that are most strongly associated with microbial community structure (Fig. 5).

## Discussion

In this study, we investigated how: (i) compost amendment, (ii) planting *B. curtipendula*, and (iii) inoculation of *B. curtipendula* with beneficial endophytes influenced the microbiome of metal-contaminated mine tailings. We found that compost amendment and endophyte inoculation synergistically increased prokaryotic diversity in the mine tailings and influenced both fungal and prokaryotic community composition, with compost amendment having a dominant effect on microbial communities (Table 2, Fig. 5). Total nitrogen, organic carbon, and water-extractable concentrations of Cu and Sb, which were significantly higher in compost-amended treatments, were identified as the variables that were most strongly associated with community structure in the mine tailings (Fig. 5, Table 1).

Compost application has been shown to promote restoration of metalliferous soils via modulation of both biotic and abiotic factors in the soil [42, 74–76]. For instance, compost amendment was shown to have a beneficial influence on revegetation of mine tailings by enhancing soil structure [77], bacterial root colonization [43], and plant biomass production [43, 77]. Here, we establish that compost amendment significantly increased prokaryotic diversity, which also corresponded to higher content of organic carbon and total nitrogen in the mine tailings (Table 1) and higher plant biomass [50],under review). Similar results were also reported in a study by Maron et al. [78], an increase of prokaryotic diversity that positively correlated with nutrient availability and organic matter transformation in soil. Furthermore, our results show that organic carbon and total nitrogen originating from compost were significantly associated with changes in the structure of both prokaryotic and fungal communities (Fig. 5). Nutrients and/or carbon and energy sources released during microbial decomposition of added compost could be used by plants and their associated microorganisms and, in turn, promote secondary succession of both plants and microorganisms in these degraded soils if applied in the field, as analogously reported by Gil-Loaiza et al. [13].

Planting *B. curtipendula* did not significantly influence microbial diversity, which is in contrast with studies that reported an increase in microbial diversity upon revegetation of mine tailings [19, 79, 80]. However, planting *B. curtipendula* did affect prokaryotic and fungal community structure in the tailings, and in the case of prokaryotes, in compost as well. The observed shift in microbial communities as a response to planting could be attributed to (i) physical changes in tailings structure induced by the roots and/or (ii) rhizodeposition and resulting enrichment of microbial populations which can use rhizodeposits as carbon and/or energy sources [81].

Several studies have demonstrated the potential of plant inoculation with beneficial endophytes to increase phytoremediation efficiency of metal-contaminated soils [82–84]. Here, we report that endophyte inoculation significantly influenced the structure of prokaryotic communities in both mine tailings and compost (Table 2) and increased prokaryotic and fungal diversity in the mine tailings. In the case of tailings, there was a significant interactive effect between the endophyte inoculation and compost addition on the prokaryotic community structure. Moreover, compost amendment and endophyte inoculation synergistically increased prokaryotic diversity in the mine tailings (Fig. 2). Surprisingly, the initial pre-treatment mine tailings had the highest prokaryotic and fungal diversity compared to any of the treated tailings post-incubation. This demonstrates that despite their hostility to plants, mine tailings harbor diverse prokaryotic and fungal communities that are well adapted to these anthropogenic soils [3, 85, 86]. The increase in moisture by regular watering and initial adjustment of pH by the addition of dolomite, which was necessary to support plant growth [47], likely caused the loss of prokaryotic taxa that were specifically adapted to dry and acidic conditions [87, 88]. The initial mine tailings were dominated by Bacillota (Fig. 3), members of this phylum are known for their ability to form endospores and survive in extreme environments [89]. Therefore, it is not surprising that they formed a majority in dry and metal-contaminated mine tailings, similarly to the findings of Khan et al. [90], Ji et al. [91]. High diversity of taxa mainly belonging to Bacillota that are generally well adapted to these unique yet hostile human-made microbial habitats does not necessarily mean that such communities would support restorative processes, including improving soil health and allowing plant colonization. In the case of fungi, the phylum Mucoromycota was found predominantly in the initial compost and represented only a minor fraction in the initial mine tailings and seeds. After 2 months of plant growth, Mucoromycota were found to be more abundant in the mine tailings amended with compost (TC, TPC, and TPEC) and in the roots (TPC and TPEC) in contrast to mine tailings without added compost (T, TP, and TPE) (Fig. 3. This confirmed the occurrence of horizontal transmission of microorganisms between the studied habitats: the mine tailings, compost, and roots. Although there were no significant differences in bacterial and fungal abundances in tailings among treatments; the fungal:bacterial ratio was significantly higher in tailings in treatments amended with compost in contrast to treatments without compost (Additional file 1: Fig. S1), which possibly reflects the spread of compost-associated fungi from the compost layer into tailings. Thus, in addition to being a source of organic carbon and total nitrogen, compost also served as a microbial inoculum. As recently argued by See et al. [92], fungal hyphae colonize mineral microsites in soil and, together with bacteria that use hyphae as routes for transmission, contribute to the deposition of organic matter onto minerals and further transformation of mineral-associated organic matter. With that in mind, measured increases in organic carbon, total nitrogen (Table 1) and prokaryotic diversity in the tailings (Fig. 2) could be related to the spread of compost-associated microorganisms into the tailings, hypothetically acting in synergy with endophytic inoculation.

Differential abundance analysis revealed that: (i) compost amendment, (ii) planting *B. curtipendula*, and (iii) endophyte inoculation, as well as combinations of these strategies, influenced community members in the mine tailings. Thirty-six bacterial genera and sixteen fungal genera were significantly enriched in the mine tailings subjected to at least one of the following treatments: TC, TP, TPE and TPEC vs control (T) (Fig. 4). While some genera were unique to a specific treatment, others were shared, and importantly, a combination of all approaches (TPEC) resulted in the highest number of enriched bacterial genera (Fig. 4A). While the endophytic inoculants were not found to be among the significantly enriched taxa, treatments with compost (TC, TPC and TPEC), which had the highest content of total nitrogen, were enriched in *Brevibacillus*, *Brevundimonas*, *Corynebacterium*, *Lysinibacillus*, *Mycobacterium*, *Rhodococcus* and *Streptomyces*. These bacterial genera have been shown to contain members that are able to fix atmospheric nitrogen [93–97] and, thus, activity of these potentially diazotrophic taxa could have contributed to the observed increase in total nitrogen in tailings amended with compost. Furthermore, changes in microbial community composition significantly corresponded to increased water-extractable concentrations of two potentially toxic trace elements: Cu and Sb, which were highest in TPEC treatment and exceeded water regulatory limits for aquatic and wildlife by at least four orders of magnitude [46] (Table 1, Fig. 5). Microorganisms have been shown to influence the mobility of trace elements in the soil through biosorption, oxidation/reduction, or complexation with siderophores and extracellular polymeric substances [98]. While it would require further investigation to reveal potential mechanisms of trace element transformation by the enriched populations in the current study, it should be noted that the activity of these taxa could be behind the increased mobilization of Cu and Sb in the mine tailings. In addition, it has been shown that higher content of dissolved organic compounds associated with compost amendment can facilitate solubilization of trace elements such as Cu via complexation [99, 100]. Thus, it is also possible that the observed increase in mobilization of Cu and Sb was mediated by changes in organic matter pools resulting from compost addition or by a joint contribution of both the abiotic and microbial processes in compost-amended tailings.

To conclude, we show that the combination of compost amendment, planting *B. curtipendula*, and endophyte inoculation (TPEC) increased prokaryotic diversity and shifted tailings microbiota composition, which significantly correlated with an improvement in soil health metrics (higher levels of total nitrogen, organic carbon, and pH). On the other hand, observed shifts in tailings microbiota due to treatments with compost also corresponded to increased mobilization of Cu and Sb, which was highest under TPEC treatment. Our study demonstrates that the initial choice of remediation strategy can cause downstream shifts in mine waste microbiome, and that an increase in microbial diversity and soil health metrics, which are often linked to functioning of healthy ecosystems and their stability [101, 102], can be accompanied by increased potential for toxic metal leaching from the mine waste which could pose a serious risk to human health. With that in mind, preliminary investigation of the responses of both biotic and abiotic factors to potential restoration strategies ex situ may help decipher how to steer microbiomes in degraded soils in a direction that is beneficial to both soil and human health and is more efficient at restoring disturbed ecosystems.

### Supplementary Information

**Additional file 1**. Supplementary data and results. **Figure S1.** qPCR data expressed as bacterial (16S rRNA) gene copy number and fungal (28S rRNA) gene copy number (log-transformed), and the ratio of bacteria:fungi for pre-treatment materials and ending treatments for both compost and tailing layers (collected after 56 days; 57 days; and 58 days of incubation in the case of TP and TPC; TPE and TPEC; and T and TC treatments, respectively). Ending treatments included tailings (T; n = 8), tailings with added compost (TC, n = 8), tailings with a plant (TP, n = 13), tailings with a plant and added compost (TPC, n = 8), tailings with a plant inoculated with endophytes (TPE, n = 11) and tailings with a plant inoculated with endophytes and added compost (TPEC, n = 8). Pre-treatment materials included initial compost (Cin, n = 4) and initial tailings (Tin, n = 6). **Table S1.** General characterization and total trace element content in pre-treatment materials: Blue Nose mine tailings amended with dolomite (Tin) and compost (Cin). **Table S2.** The plant growth-promoting properties of endophytes used for the inoculation of B. curtipendula seeds. **Table S3.** Pairwise comparison of prokaryotic (a) and fungal (b) Shannon diversity indices in tailings, compost, and roots between treatments. **Table S4.** Pairwise comparison of prokaryotic (a) and fungal (b) community structure in tailings, compost, and roots of B. curtipendula between treatments.

## Data Availability

The sequence data supporting the conclusions of this article are available in NCBI Short Read Archive under the accession number PRJNA697926. The datasets including chemical characteristics of mine tailings are available at 10.5066/P9M2JW70 and 10.5066/P99OYEXQ. All sc ripts used for analyses in R are available at the authors' GitHub repository (https://github.com/martinafarren/Phytostabilization_experiment).
