# Supplementary material for: Compost, plants and endophytes versus metal contamination: choice of a restoration strategy steers the microbiome in polymetallic mine waste
Source: Environ Microbiome. 2023 Oct 7;18:74. doi: 10.1186/s40793-023-00528-3 (PMC10559404; doi:10.1186/s40793-023-00528-3)
Supplement: Supplementary file 1 — Additional file 1. Supplementary data and results. Figure S1. qPCR data expressed as bacterial (16S rRNA) gene copy number and fungal (28S rRNA) gene copy number (log-transformed), and the ratio of bacteria:fungi for pre-treatment materials and ending treatments for both compost and tailing layers (collected after 56 days; 57 days; and 58 days of incubation in the case of TP and TPC; TPE and TPEC; and T and TC treatments, respectively). Ending treatments included tailings (T; n = 8), tailings with added compost (TC, n = 8), tailings with a plant (TP, n = 13), tailings with a plant and added compost (TPC, n = 8), tailings with a plant inoculated with endophytes (TPE, n = 11) and tailings with a plant inoculated with endophytes and added compost (TPEC, n = 8). Pre-treatment materials included initial compost (Cin, n = 4) and initial tailings (Tin, n = 6). Table S1. General characterization and total trace element content in pre-treatment materials: Blue Nose mine tailings amended with dolomite (Tin) and compost (Cin). Table S2. The plant growth-promoting properties of endophytes used for the inoculation of B. curtipendula seeds. Table S3. Pairwise comparison of prokaryotic (a) and fungal (b) Shannon diversity indices in tailings, compost, and roots between treatments. Table S4. Pairwise comparison of prokaryotic (a) and fungal (b) community structure in tailings, compost, and roots of B. curtipendula between treatments. [file 40793_2023_528_MOESM1_ESM.docx]

**SUPPLEMENTARY MATERIAL**

Compost, plants and endophytes versus metal contamination: Choice of a restoration strategy steers the microbiome in polymetallic mine waste

Martina Kracmarova-Farren^1*^, Jakub Papik^1*^ ^✉^, Ondrej Uhlik^1^, John Freeman^2^, Andrea Foster^3^, Mary-Cathrine Leewis^3,4^, Courtney Creamer^3^

*^1^University of Chemistry and Technology, Prague, Faculty of Food and Biochemical Technology, Department of Biochemistry and Microbiology, Prague, Czech Republic*

*2 Intrinsyx Environmental, Sunnyvale, CA, 94085*

*^3^U.S. Geological Survey, Menlo Park, California*

*^4^Agriculture and Agri-Food Canada, Quebec Research and Development Centre, Quebec, QC, Canada*

^✉^**Corresponding author:** University of Chemistry and Technology, Prague, Technicka 3, 166 28 Prague 6, Czech Republic. Phone: +420 220 44 5136; email: jakub.papik@vscht.cz

*Both authors contributed equally to the study.

**Figure S1.** qPCR data expressed as bacterial (16S rRNA) gene copy number and fungal (28S rRNA) gene copy number (log-transformed), and the ratio of bacteria:fungi for pre-treatment materials and ending treatments for both compost and tailing layers (collected after 56 days; 57 days; and 58 days of incubation in the case of TP and TPC; TPE and TPEC; and T and TC treatments, respectively). Ending treatments included tailings (T; n = 8), tailings with added compost (TC, n = 8), tailings with a plant (TP, n = 13), tailings with a plant and added compost (TPC, n = 8), tailings with a plant inoculated with endophytes (TPE, n = 11) and tailings with a plant inoculated with endophytes and added compost (TPEC, n = 8). Pre-treatment materials included initial compost (C_in_, n = 4) and initial tailings (T_in_, n = 6).

**Table S1**. General characterization and total trace element content in pre-treatment materials: Blue Nose mine tailings amended with dolomite (T_in_) and compost (C_in_).

|  | **Tin** | **Cin** | |
| --- | --- | --- | --- |
| pH | 5.11 | | 6.84 |
| Water holding capacity (g/g) | 0.41 | | 1.73 |
| Surface area (m^2^/g) | 5.97 | | - |
| Organic carbon (mg/g) | 2.00 | | 298.72 |
| Inorganic carbon (mg/g) | 4.70 | | 0.00 |
| Total nitrogen (mg/g) | 0.079 | | 15.802 |
| Dissolved organic carbon (µg/g) | 9.27 | | 2932.21 |
| Microbial biomass (µg/g) | 4.14 | | 1931.94 |
| As (µg/g) | 97 | | - |
| Cd (µg/g) | 4.5 | | - |
| Co (µg/g) | 2.6 | | - |
| Cu (µg/g) | 253 | | - |
| Mn (µg/g) | 793 | | - |
| Pb (µg/g) | 15667 | | - |
| Sb (µg/g) | 316 | | - |
| Zn (µg/g) | 1127 | | - |

## These data were published in Creamer et al. (2022a) and full data sets are available for download from Creamer et al (2022b) (US Geological Survey Science Base Repository Data Release: doi.org/10.5066/P99OYEXQ).

**Table S2**. The plant growth-promoting properties of endophytes used for the inoculation of B. curtipendula seeds.

| **Nearest neighbour** | **N-fixation** | **P solubilization** | **GenBank ID** | **Source** | **Original study** |
| --- | --- | --- | --- | --- | --- |
| *Pseudomonas* sp*.* | + | + | KU557506 | *Salix sitchensis*; stem | Doty et al. (2009) |
| *Curtobacterium* sp. | + | + | KU523564 | *Salix sitchensis*; stem | Doty et al. (2009) |
| *Burkholderia* sp. | + | + | KU523562 | *Populus trichocarpa*; stem | Doty et al. (2009) |
| *Acinetobacter* sp. | + | + | KU523563 | *Populus trichocarpa*; stem | Doty et al. (2009) |
| *Rhodotorula* sp*.* | + | + | EU563924.1 | *Populus trichocarpa*; stem | Firrincieli et al. (2015) |
| *Burkholderia* sp. | + | + | KF597276 | *Populus trichocarpa*; stem | Kandel et al. (2017) |
| *Rahnella* sp. | + | + | KU497675 | *Populus trichocarpa*; stem | Doty et al. (2009) |
| *Sphingomonas* sp*.* | + | + | KT984987 | *Salix sitchensis*; stem | Doty et al. (2009) |
| *Rhizobium* sp*.* | + | + | KT 962907 | *Populus trichocarpa* x deltoides; stem | Doty et al. (2005) |
| *Herbaspirillum* sp. | + | unknown | KU495919 | *Salix sitchensis*; stem | Doty et al. (2009) |

**Table S3.** Pairwise comparison of prokaryotic (a) and fungal (b) Shannon diversity indices in tailings, compost, and roots between treatments.

| **Tailings** |  | T_in_ | T | TP | TPE | TC | TPC |
| --- | --- | --- | --- | --- | --- | --- | --- |
| a) prokaryotes | T | **0.0047** | - | - | - | - | - |
|  | TP | **0.0016** | 0.2500 | - | - | - | - |
|  | TPE | **0.0026** | **0.0041** | **0.0001** | - | - | - |
|  | TC | **0.0047** | **0.0013** | **0.0001** | **0.0047** | - | - |
|  | TPC | **0.0047** | **0.0004** | **0.0001** | **0.0001** | 0.0551 | - |
|  | TPEC | **0.0047** | **0.0004** | **0.0001** | **0.0001** | **0.0004** | 0.2786 |
| b) fungi | T | **0.0166** | - | - | - | - | - |
|  | TP | **0.0031** | 0.2154 | - | - | - | - |
|  | TPE | **0.0166** | 0.5043 | **0.0343** | - | - | - |
|  | TC | **0.0061** | 0.1809 | 0.8665 | **0.0343** | - | - |
|  | TPC | **0.0047** | 0.1142 | 0.8665 | **0.0343** | 0.8665 | - |
|  | TPEC | **0.0042** | 0.5608 | 0.2154 | 0.1142 | 0.1149 | 0.0575 |
| **Compost** |  | C_in_ | TC | TPC |  |  |  |
| a) prokaryotes | TC | **0.0121** | - | - |  |  |  |
|  | TPC | **0.0084** | 0.6806 | - |  |  |  |
|  | TPEC | **0.0084** | 0.4487 | 0.5873 |  |  |  |
| b) fungi | TC | **0.0081** | - | - |  |  |  |
|  | TPC | **0.0081** | 0.9778 | - |  |  |  |
|  | TPEC | **0.0081** | 0.9778 | 1.0000 |  |  |  |
| **Roots** |  | S_in_ | TPC |  |  |  |  |
| a) prokaryotes | TPC | **0.0360** | - |  |  |  |  |
|  | TPEC | **0.0290** | 0.4000 |  |  |  |  |
| b) fungi | TPC | 0.0710 | - |  |  |  |  |
|  | TPEC | 0.1710 | 0.0860 |  |  |  |  |

Treatments: tailings (T), tailings with added compost (TC), tailings with a plant (TP), tailings with a plant and added compost (TPC), tailings with a plant inoculated with endophytes (TPE) and tailings with a plant inoculated with endophytes and added compost (TPEC). Initial materials included initial tailings (T_in_), compost (C_in_) and seeds (S_in_). Signiﬁcant *p* values (*p_adj_* ≤ 0.05, Pairwise Wilcoxon rank sum test) are underlined and shown in bold.

**Table S4.** Pairwise comparison of prokaryotic (a) and fungal (b) community structure in tailings, compost, and roots of *B. curtipendula* between treatments.

| Tailings: | **a) prokaryotes** | | **b) fungi** | |
| --- | --- | --- | --- | --- |
|  | R^2^ | *p_adj_* | R^2^ | *p_adj_* |
| T vs TP | 0.374 | **0.0002** | 0.293 | **0.0002** |
| T vs TPE | 0.242 | **0.0002** | 0.334 | **0.0002** |
| T vs TC | 0.530 | **0.0003** | 0.587 | **0.0003** |
| T vs TPC | 0.470 | **0.0002** | 0.562 | **0.0002** |
| T vs TPEC | 0.590 | **0.0002** | 0.567 | **0.0002** |
| TP vs TC | 0.527 | **0.0002** | 0.555 | **0.0002** |
| TC vs TPC | 0.186 | **0.0002** | 0.097 | 0.1331 |
| TP vs TPC | 0.539 | **0.0002** | 0.541 | **0.0003** |
| TC vs TPE | 0.606 | **0.0002** | 0.610 | **0.0003** |
| TP vs TPE | 0.488 | **0.0002** | 0.095 | **0.0030** |
| TPC vs TPE | 0.545 | **0.0002** | 0.587 | **0.0002** |
| TC vs TPEC | 0.301 | **0.0002** | 0.147 | **0.0003** |
| TP vs TPEC | 0.651 | **0.0002** | 0.538 | **0.0002** |
| TPC vs TPEC | 0.269 | **0.0005** | 0.060 | 0.4892 |
| TPE vs TPEC | 0.670 | **0.0002** | 0.583 | **0.0002** |
| Compost: | **a) prokaryotes** | | **b) fungi** | |
|  | R^2^ | *p_adj_* | R^2^ | *p_adj_* |
| TC vs TPC | 0.170 | **0.0001** | 0.042 | 0.7473 |
| TC vs TPEC | 0.185 | **0.0001** | 0.046 | 0.7473 |
| TPC vs TPEC | 0.174 | **0.0001** | 0.045 | 0.7473 |
| Plant roots: | **a) prokaryotes** | | **b) fungi** | |
|  | R^2^ | *p_adj_* | R^2^ | *p_adj_* |
| TPC vs TPEC | 0.221 | 0.1714 | 0.151 | 0.4286 |

Treatments: tailings (T), tailings with added compost (TC), tailings with a plant (TP), tailings with a plant and added compost (TPC), tailings with a plant inoculated with endophytes (TPE) and tailings with a plant inoculated with endophytes and added compost (TPEC). Signiﬁcant *p* values (*p_adj_* ≤ 0.01, Pairwise PERMANOVA) are underlined and shown in bold.

**References**

Creamer, C.A., Leewis, M.-C., Governali, F.C., Freeman, J.L., Gray, F., Wright, E.G., Foster, A.L., 2022a. Microbial endophytes and compost improve plant growth in two contrasting types of hard rock mining waste. Int J Phytoremediation, 1-8.

Creamer, C.A., Leewis, M.C., Wright, E., and Foster, A., 2022b. Grass Growth in Mining Wastes with Compost and Endophyte Additions: U.S. Geological Survey data release, doi.org/[10.5066/P99OYEXQ](https://doi.org/10.5066/P99OYEXQ).

Doty, S.L., Dosher, M.R., Singleton, G.L., Moore, A.L., Van Aken, B., Stettler, R.F., Strand, S.E., Gordon, M.P., 2005. Identification of an endophytic Rhizobium in stems of Populus. Symbiosis.

Doty, S.L., Oakley, B., Xin, G., Kang, J.W., Singleton, G., Khan, Z., Vajzovic, A., Staley, J.T., 2009. Diazotrophic endophytes of native black cottonwood and willow. Symbiosis 47, 23-33.

Kandel, S.L., Firrincieli, A., Joubert, P.M., Okubara, P.A., Leston, N.D., McGeorge, K.M., Mugnozza, G.S., Harfouche, A., Kim, S.-H., Doty, S.L., 2017. An in vitro study of bio-control and plant growth promotion potential of Salicaceae endophytes. Front Microbiol 8, 386.

Firrincieli, A., Otillar, R., Salamov, A., Schmutz, J., Khan, Z., Redman, R.S., Fleck, N.D., Lindquist, E., Grigoriev, I.V., Doty, S.L., 2015. Genome sequence of the plant growth promoting endophytic yeast Rhodotorula graminis WP1. Front Microbiol 6, 978.
